# Profiling chronic migraine patients according to clinical characteristics: a cluster analysis approach

**DOI:** 10.3389/fneur.2025.1569333

**Published:** 2025-03-10

**Authors:** Masahito Katsuki, Yasuhiko Matsumori, Shin Kawamura, Kenta Kashiwagi, Akihito Koh, Tetsuya Goto, Kazuma Kaneko, Naomichi Wada, Fuminori Yamagishi

**Affiliations:** ^1^Insight Science Foundation Ireland Research Centre for Data Analytics, School of Human and Health Performance, Dublin City University, Dublin, Ireland; ^2^Physical Education and Health Center, Nagaoka University of Technology, Niigata, Japan; ^3^Department of Neurosurgery, Suwa Red Cross Hospital, Suwa, Japan; ^4^Headache Outpatient, Suwa Red Cross Hospital, Suwa, Japan; ^5^Sendai Headache and Neurology Clinic, Sendai, Japan; ^6^Department of Neurosurgery, Itoigawa General Hospital, Itoigawa, Japan; ^7^Department of Neurology, Itoigawa General Hospital, Itoigawa, Japan; ^8^Department of Neurology, Suwa Red Cross Hospital, Suwa, Japan; ^9^Department of Surgery, Itoigawa General Hospital, Itoigawa, Japan

**Keywords:** chronic migraine, cluster analysis (clustering analysis), data-driven medicine, medication-overuse headache, tension-type headache

## Abstract

**Background:**

To group the characteristics of chronic migraine (CM) by headache characteristics.

**Methods:**

We performed a retrospective analysis of the medical records of 821 adult CM patients who visited a specialized outpatient clinic for headaches. Using the headache characteristics, we performed Density-Based Spatial Clustering of Applications with Noise (DBSCAN) clustering to group CM patients. The burdens to their lives, monthly headache days (MHD), monthly acute medication intake days (AMD), and treatment outcomes were evaluated among the clusters.

**Results:**

Through a cluster analysis based on headache characteristics, our findings indicated the potential existence of three distinct types of CM: cluster 1 (predominantly female with CM resembling migraine), cluster 2 (higher age, higher BMI, smoker), and cluster 3 (mostly female with CM that have fewer migraine characteristics). The impact on quality of life was significant in cluster 1 compared to cluster 3. However, there were no differences in treatment outcomes, initial MHD, AMD, the years of migraine, or treatment sensitivity among these three clusters.

**Conclusion:**

Cluster analysis mathematically divided CM patients into three groups, with predominant differences in the degree of disruption to their lives and their characteristics; further research is needed on the diagnostic criteria for CM and its characteristics.

## 1 Introduction

Chronic migraine (CM) is defined in the International Classification of Headache Disorders, 3rd edition (ICHD-3) as having headaches on at least 15 days per month, with eight of these having migraine symptoms, for at least 3 months. CM exerts significant negative effects on a patient’s life, severely impacting socioeconomic functioning and overall quality of life (1). It is observed in 1–2% of the general population and about 8% of migraine patients (2), often arising from episodic migraine at an annual conversion rate of approximately 3% (3). The process of becoming chronic is potentially reversible, as around 26% of CM patients experience remission within 2 years of the onset (2). The primary modifiable risk factors contributing to CM involve the excessive use of acute medication, ineffective acute treatment, obesity, depression, and stressful life events. Additionally, age, and being female elevate the risk of transitioning into CM (4).

Broadening the CM definition from ICHD-2 to ICHD-3 has brought important consequences. It now acknowledges varying migraine attack severity, even milder ones resembling tension-type headache (TTH) due to lacking migraine features. As a result, studies based on the new criteria by ICHD-3 may examine a different population both clinically and physiologically compared to earlier research (4). Also, the definition of CM itself is still under debate. Ishii et al. have argued that the definition of 15 monthly headache days (MHD) does not necessarily match the severity of the patient’s headache and its interference with life, and that it may underdiagnose patients with CM (5). CM is a heterogeneous group of patients, yet its classification definitions and clinical characteristics are not definitive.

Cluster analysis is a method of mathematically classifying a subject by gathering together those similar to each other from a group of different things to form a settlement (cluster). There have been previous attempts to reclassify headaches by cluster analysis (6–11), which may provide new insights that are not based on ICHD-3. Only one report (11) has attempted to classify especially CM, using 14 clinical variables. To reconsider the characteristics of patients with CM, we herein perform cluster analysis based on the headache characteristics for the CM patients diagnosed by a headache specialist, and investigate the relationship between the subtypes, burden, and prognosis. The previous study (11) used clustering for comprehensive variables, including both headache characteristics and mainly psychological burden scales. On the other hand, our study is novel in that we first clustered patients based solely on their baseline and headache characteristics and then compared the differences in headache burdens and treatment outcomes between clusters.

## 2 Materials and methods

### 2.1 Study population

From the medical records between January 2020 and December 2022, we retrospectively investigated 6,058 consecutive first-visit headache patients who presented at the headache-specialized outpatient. After a medical and appropriate radiological examination, a headache specialist diagnosed all patients based on the ICHD-3 criteria. Diagnosis with CM (ICHD code 1.3) and age 15 or over were the inclusion criteria. This is because the Japanese Pharmaceutical Affairs Law considers persons 15 years of age and older to be adults. This dataset was partially used for a previous study (12).

Using the headache questionnaire sheet at the first consultation, we retrieved variables as follows: age, sex, height, weight, dominant hand, presence of habitual drinking, smoking, bedtime, wake-up time, age at headache onset, headache frequency, headache duration, site of headache, headache characteristics, headache severity, presence of headache aggravation and its cause, concomitant symptoms, presence of aura, times when the headache is most likely to occur, inducement of headache, use of acute medication, and family history. The patients completed the questionnaire, and no missing values were found because nurses assisted them in answering questionnaires as needed. The patients were instructed as “Please write about your headaches for the past 3 months.” The items in the questionnaire are listed in Table 1. In addition to the questionnaire sheets, the headache-specialized doctor asked the patients about their headaches and burdens in detail.

**Table 1:** Headache questionnaire sheet

Questions	Answers
01. Age	( ) y.o.
02. Sex	Male/Female
03. Height	( ) cm
04. Weight	( ) kg
05. Dominant hand	Right, Left, Other ( )
06. Habitual drinking	No, Sometimes, Everyday
07. Habitual smoking	No, Previous, Current
08. Bedtime	AM/PM ( : )
09. Wake up time	AM/PM ( : )
10. Headache onset age	( ) y.o., ( ) days/months/years ago
11. Headache frequency	( ) times per minute/h/month/year, Every day
12. Headache duration	Always, ( ) days, One day, Half a day, ( ) hours, ( ) minutes, Moment
13. Site of headache	Unilateral (right/left), Bilateral, Center, Different site, Around the eye, Front, Back, Side, Top, Craniocervical transitional, Ear, Chin, Nose, Teeth
14. Headache characteristics	Pulsating, Constricting, Stabbing, Tingling, Grasped, Gouged out, Racking, Dull, Others ( )
15. Headache severity	Numerical Rating Scale ( /10) Needs rest, Disturbing daily life without rest, Not disturbing
16. Presence of aggravation or improvement by exercise	Aggravation, Improvement, No change
17. Concomitant symptoms	Nausea, Vomiting, Photophobia, Phonophobia, Osmophobia, Red eye, Lacrimation, Runny nose, Dizziness, Fatigue, Stiff shoulders, Numbness in the extremities, Others ( )
18. Presence of prodrome	Absent, Scintillating scotoma, Numbness in the extremities, Increased appetite, Edema, Sleepy, Frequent urination, Nausea, Vomiting, Photophobia, Phonophobia, Osmophobia
19. Times when headaches are most likely to occur	Wake up, Morning, Afternoon, Evening, Sleeping
20. Inducement of headache	None, Lack of sleep, Too much sleep, Tired, Drinking, Smoking, Bathing, Weather, Light, Loudness, Smell, Holiday, Crowd, Weather
21. Use of acute medication	Drug’s name: ( ) Frequency: ( ) times per day/ month/year Effectiveness: Very effective, Mild effective, Not effective, Different at the times
22. Does anybody in your family have headaches?	Yes/No (Mother, Father, Son, Daughter, Grandmother, Grandfather, Brother, Sister)
23. Other scales related to headaches.	Headache Impact Test-6 (HIT-6), Generalized Anxiety Disorder-7 (GAD-7), Patient Health Questionnaire-9 (PHQ-9), Migraine Disability Assessment Scale (MIDAS), 12-item Allodynia Symptom Checklist (ASC-12).

Ask patients to check or fill out each item on the questionnaire.

From the medical record, we investigated MHD, monthly acute medication days (AMD), Headache Impact Test-6 (HIT-6), Generalized Anxiety Disorder-7 (GAD-7), Patient Health Questionnaire-9 (PHQ-9), Migraine Disability Assessment Scale (MIDAS), and 12-item Allodynia Symptom Checklist (ASC-12) at the first visit.

Patients were treated according to the Clinical Practice Guideline for Headache Disorders 2021. Valproic acid, amitriptyline, propranolol, lomerizine, verapamil, and calcitonin gene-related peptide (CGRP)-related monoclonal antibodies can be used as prophylactic medication in Japan. These prophylactic medications were started at the initial visit and continued for at least 1 month. As treatment outcomes, MHD and AMD at one, three, six, 12, and 24 months were investigated using the patients’ headache diaries.

### 2.2 Cluster analysis

Using the variables regarding physical and headache characteristics, we performed Density-Based Spatial Clustering of Applications with Noise (DBSCAN) clustering to classify the mathematic subgroups of CM patients. The number of clusters was decided (13) first based on the elbow chart and then considering clinical understandability, the silhouette score, and the Davies-Bouldin Index. To visualize high-dimensional data, we used t-distributed Stochastic Neighbor Embedding (t-SNE) to embed higher dimensional points into lower dimensions to reflect the patient similarity. Finally, the differences of variables among each cluster were investigated.

DBSCAN was chosen for several reasons. Firstly, the dataset has more variables than samples, making non-hierarchical clustering more suitable to manage computational complexity. Secondly, DBSCAN supports hard clustering, ensuring each patient is assigned to a distinct cluster, which is crucial for clinical purposes requiring clear groupings. Thirdly, hierarchical clustering is avoided due to its susceptibility to ‘chaining’ effects and sensitivity to outliers. Additionally, DBSCAN can handle non-linear relationships between variables and clusters with varying densities, which makes it more flexible compared to methods assuming spherical clusters with equal densities. This is important for handling complex data structures. Prior to applying DBSCAN, a sensitivity analysis using t-SNE was conducted to visualize and confirm the validity of the cluster structures.

### 2.3 Statistical analysis

To understand the characteristics of each cluster, we compared the centroids of each cluster, MHD, AMD, HIT-6, GAD-7, PHQ-9, MIDAS, ASC-12 at the first visit, and the treatment outcomes. The Shapiro–Wilk test was used to check the normal distribution, and results are shown as mean and standard deviation (SD) for the variables with normal distribution and median (interquartile range; IQR) for those with non-normal distributions and non-continuous variables. Each variable in the questionnaire sheets or headache characteristics was treated as a nominal variable, with 1 for presence and 0 for absence. Chi-square, Fisher exact, Spearman’s correlation coefficient, or Mann–Whitney U tests were performed to compare the two groups. Finally, the Kruskal–Wallis test and subsequent Dunn test with Bonferroni correction (*p* < 0.000138/3) were used to compare the characteristics among the multiple clusters. A two-tailed *p* < 0.000138 (0.05/363) was defined as statistically significant because we performed univariate analysis 363 times: 281 items in Table 2 (full in Supplementary Table 1), 80 items in Supplementary Table 2, and 2 times in Supplementary Table 3.

**Table 2:** Characteristics of patients and each cluster.

Variables	Cluster 1 *n* = 398 (48.5%)	SD or IQR	Cluster 2 *n* = 142 (17.3%)	SD or IQR	Cluster 3 *n* = 281 (34.2%)	SD or IQR	Total *n* = 821	SD or IQR	*p* value	Subsequent analysis
Patients and headache characteristics at initial concultation										
Age at the first visit (years old)	33.4	11.7	37.4	10.2	33.3	12.3	34.1	11.7	<0.000138*	2 > 1, 2 > 3
Sex, Female as 1	89.7%		50.0%		82.9%		80.5%		<0.000138*	2 < 1, 2 < 3
Height (cm)	158.9	6.2	166.6	8.1	159.6	6.8	160.5	7.3	<0.000138*	2 > 1, 2 > 3
Weight (kg)	53.2	7.3	76.5	11.0	53.7	7.8	57.4	12.0	<0.000138*	2 > 1, 2 > 3
Body mass index	21.1	2.8	27.7	4.3	21.1	2.8	22.2	4.0	<0.000138*	2 > 1, 2 > 3
Smoking none;0 yes;1	46.0%		81.7%		36.3%		48.8%		<0.000138*	2 > 1, 2 > 3
Age of headache onset	7.5	10.5	12.2	12.8	10.3	11.4	9.3	11.4	<0.000138*	2 > 1, 2 > 3
Characteristics; throbbing “Japanese onomatopoeia, *zukin*”	77.1%		75.4%		60.1%		71.0%		<0.000138*	1 > 3
Characteristics; gradually worsen	39.7%		28.2%		21.7%		31.6%		<0.000138*	1 > 3
Visual analogue scale of pain (0–100 points)	81.0	10.4	67.1	11.9	50.9	9.1	68.3	17.0	<0.000138*	1 > 2 > 3
Interference to life; if you concentrate, you will forget the headache.	7.5%		18.3%		23.1%		14.7%		<0.000138*	3 > 1
Impediments to daily life; The pain is intense even when lying down	32.4%		22.5%		9.3%		22.8%		<0.000138*	1 > 3
Exacerbating factor; dazzling light	47.0%		34.5%		30.3%		39.1%		<0.000138*	1 > 3
Exacerbating factor; smell or odor	33.4%		21.1%		18.5%		26.2%		<0.000138*	1 > 3
Accompanied symptoms; nausea	78.6%		66.9%		56.6%		69.1%		<0.000138*	1 > 3
Accompanied symptoms; vomiting	33.4%		26.8%		15.3%		26.1%		<0.000138*	1 > 3
Accompanied symptoms; photophobia	47.7%		32.4%		32.4%		39.8%		<0.000138*	1 > 3
Usually, you use OTC analgesics.	72.1%		74.7%		75.8%		73.8%		0.543	N.P.
Usually, you use prescribed analgesics	31.9%		31.7%		19.9%		27.8%		0.00144	N.P.
Usually, you use triptans	15.3%		10.6%		4.3%		10.7%		<0.000138*	1 > 3
Burdens for lives (median and IQR)										
GAD-7 total	7	5	7	5	6	5	6	7	0.025	N.P.
PHQ-9 total	8	6	7	5	7	5	7	8	0.038	N.P.
ASC-12	1	2	1	1	1	2	1	2	0.001	N.P.
Sum of HIT-6	65	7	64	6	61	7	63	7	<0.000138*	1 > 3
MIDAS score	33	52	30	49	21	47	30	30	<0.000138*	1 > 3
Treatment outcome										
MHD at the first visit	22.4	6.2	22.5	6.0	22.4	6.2	22.4	6.1	0.852	N.P.
MHD at 3 months (*n* = 457, 55.7%)	13.2	13.4	12.6	8.0	11.8	7.9	12.7	11.0	0.589	N.P.
Difference of MHD compared to baseline at 3 months (*n* = 457, 55.7%)	−9.4	13.5	−10.1	8.5	−11.1	9.0	−10.1	11.3	0.399	N.P.
50%MHD RR at 3 months (*n* = 457, 55.7%)	61.4%		58.9%		66.7%		62.6%		0.433	N.P.
75%MHD RR at 3 months (*n* = 457, 55.7%)	24.7%		28.9%		29.2%		26.9%		0.571	N.P.
100%MHD RR at 3 months (*n* = 457, 55.7%)	2.2%		1.1%		0.7%		1.5%		0.468	N.P.
AMD at the first visit	16.4	7.8	15.9	7.7	15.5	7.7	16.0	7.7	0.253	N.P.
AMD at 3 months (*n* = 457, 55.7%)	6.5	4.8	6.9	6.1	6.1	5.0	6.4	5.1	0.641	N.P.
Difference of AMD compared to baseline at 3 months (*n* = 457, 55.7%)	−10.5	7.9	−9.9	8.1	−9.9	9.5	−10.2	8.5	0.680	N.P.
50%AMD RR at 3 months (*n* = 457, 55.7%)	79.8%		77.8%		79.9%		79.4%		0.911	N.P.
75%AMD RR at 3 months (*n* = 457, 55.7%)	35.0%		48.9%		43.8%		40.5%		0.048	N.P.
100%AMD RR at 3 months (*n* = 457, 55.7%)	4.9%		7.8%		7.6%		6.4%		0.481	N.P.

Each variable was treated as a nominal variable, with 1 for presence and 0 for absence. Mean and SD are shown for continuous variables. Median and IQR are shown for non-continuous or ordinal variables. Abbreviations; AMD; monthly acute medication intake days, ASC-12; 12-item Allodynia Symptom Checklist, GDA-7; Generalized Anxiety Disorder-7, HIT-6; Headache Impact Test-6, IQR; Interquartile range, MHD; monthly headache days, MIDAS; Migraine Disability Assessment Scale, N.P.; not performed for subsequent analysis. OTC; over-the-counter, PHQ-9; Patient Health Questionnaire-9, RR; responder rate.

A two-tailed *p* < 0.000138 (0.05/362) was defined as statistically significant because we performed statistical analysis 362 times in this manuscript.

Because this was a retrospective investigation, no statistical sample size was designed *a priori*. This is the primary *post hoc* analysis of these data. We used SPSS 28.0.0, Python 3.9.0, PyCaret 3.0.0, scikit-learn 1.3.0, and Matplotlib 3.5.1.

### 2.4 Ethical aspects

The Itoigawa General Hospital Ethics Committee approved this study (approval number: 2022–2, 2022–10). The requirement for written informed consent was waived because of the study’s retrospective nature. Opt-out consent documents were presented on the Itoigawa General Hospital website^1^ and Sendai Headache and Neurology Clinic for patients who did not wish to participate. Informed consent was waived, but participants completed the questionnaire in the usual clinical practice. All methods were performed following the Strengthening the Reporting of Observational Studies in Epidemiology guidelines and regulations of the Declaration of Helsinki.

## 3 Results

### 3.1 General characteristics

Of 6,058 patients’ medical records, who were all Asian, 821 (13.6%) were diagnosed with CM and aged 15 or older. The mean age was 34.1 (SD 11.7) years, and 661 (80.5%) were female. The mean MHD was 22.4 (SD 6.1) days, and the mean AMD was 16.0 (SD 7.4) days. Medication overuse was accompanied by 434 (52.9%) patients. The mean years of disease was 18.2 years (SD 11.3). The median HIT-6 was 63 (IQR 7), the median GAD-7 6 (7), PHQ-9 7 (8), MIDAS 30 (50), and ASC-12 1 (2). All patients were not prescribed prophylactic medications before the first visit.

After diagnosis of CM, prophylactic medications were started at the initial visit and continued for at least 1 month. Of the 821 patients, 132 (16.1%) did not receive prophylactic medication at the initial consultation, but 641 (78.1%) patients were prescribed one prophylactic medication, 45 (5.5%) had two, and 3 (0.4%) had three (Table 3).

**Table 3:** Prophylactic treatment choice.

	Prophylactic treatment^†^					Number of prophylaxes			
Cluster	Lomerizine	Propranolol	Valproate	Amitriptyline	CGRP-related antibody	None	One	Two	Three or more
1 (*n* = 398)	34	7	259	52	17	61	310	25	2
2 (*n* = 142)	8	4	91	26	10	20	112	9	1
3 (*n* = 281)	14	8	174	42	3	51	219	11	0

^†Allow duplicate selection of medications; CGRP, calcitonin gene-related peptide.^

As treatment outcome, MHD and AMD at 1 month (*n* = 561, 68.3%) were 15.9 (8.0) and 7.6 (5.4) days, those at 3 months (*n* = 457, 55.7%) were 12.7 (11.0) and 6.4 (5.1) days, those at 6 months (*n* = 378, 46.0%) were 10.2 (7.4) and 5.4 (3.9) days, those at 12 months (*n* = 300, 36.5%) were 8.2 (6.8) and 5.1 (4.6) days, and those at 24 months (*n* = 177, 21.6%) were 6.8 (6.2) and 4.7 (4.7) days, respectively (Table 2; Supplementary Table 1).

### 3.2 Clustering results

**Figure 1:**
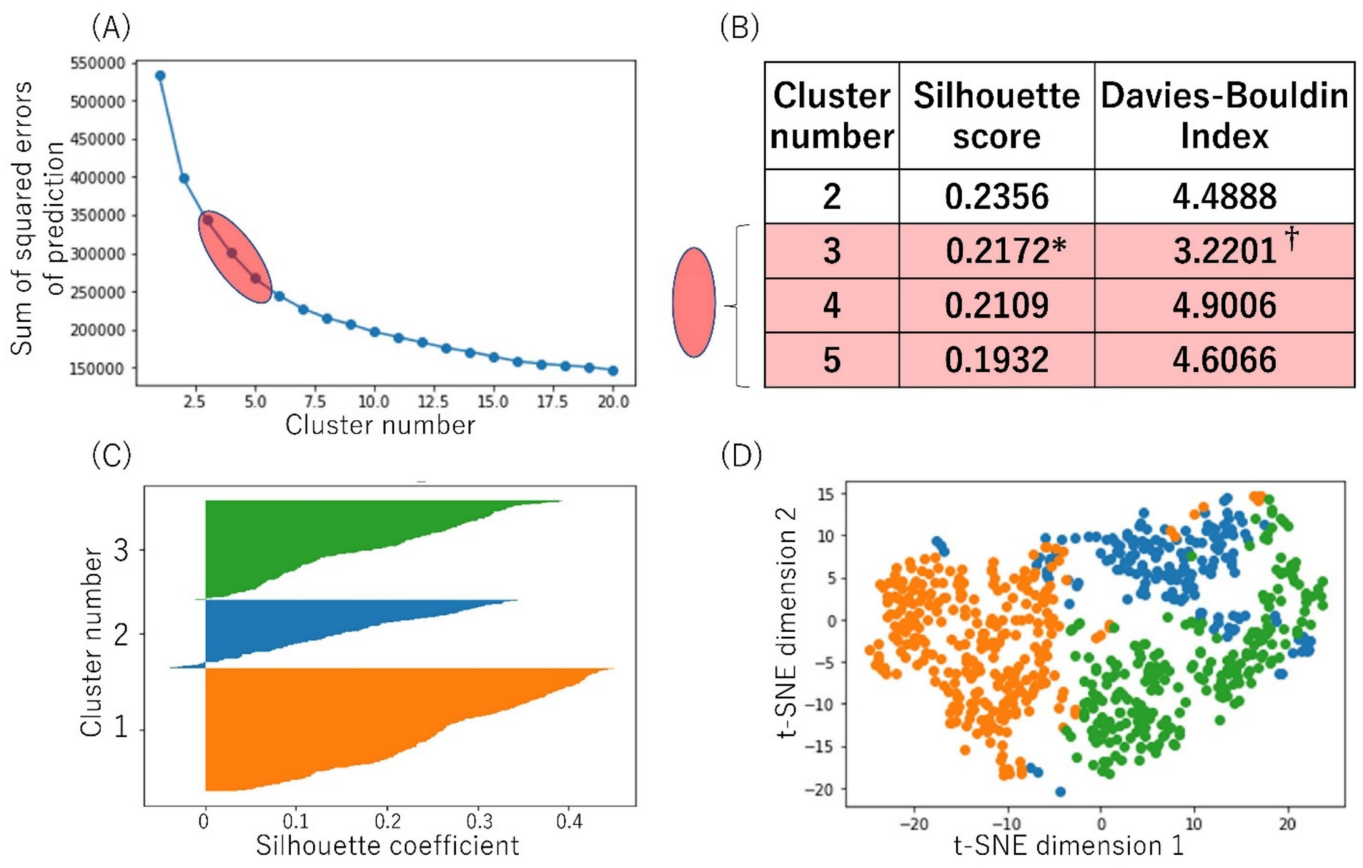
Cluster analysis. **(A)** The elbow chart calculated by DBSCAN, suggesting 3–5 is the appropriate number of clusters. **(B)** The silhouette score was calculated, and that of the 3 clusters was highest at 0.2172* in cluster 3 to cluster 5. The Davies-Bouldin index of 3 clusters was lowest at 3.2201†. **(C)** Silhouette plot. **(D)** t-SNE plot. DBSCAN; Density-Based Spatial Clustering of Applications with Noise, t-SNE; t-distributed Stochastic Neighbor Embedding.

We then performed cluster analysis using DBSCAN. The elbow chart suggested 3 to 5 is the appropriate number of clusters (Figure 1A). The silhouette score of 3 clusters was highest at 0.2172. The Davies-Bouldin index of 3 clusters was lowest at 3.2201 (Figure 1B). Therefore, we decided the number of clusters as 3. Silhouette plot and t-SNE plot were shown in (Figures 1C,D). The other silhouette plots of different cluster numbers are shown in Supplementary Figure 1.

The number of patients in each cluster is 398 patients in cluster 1, 142 in cluster 2, and 281 in cluster 3, respectively. The centroids of each cluster are shown in Table 2; only those significantly different items in the Kruskal-Wallis test are shown in Table 2, and all results are shown in Supplementary Table 1. The years of disease were not different among the clusters. The age at the initial visit, height, weight, body mass index (BMI), and the age of headache onset of cluster 2 were higher than those of clusters 1 and 3. BMI was compared in each cluster by biological sex to avoid confounding, and cluster 2 had a higher BMI than clusters 1 and 3, even when sex was separated (Supplementary Table 3). Smoking was frequent in cluster 2 compared to clusters 1 and 3. The headache characteristics of cluster 2 were not statistically different from those of clusters 1 and 3. Throbbing characteristics (*zukin* in Japanese onomatope), gradually worsening characteristics, the impediments to daily life” The pain is intense even when lying down,” exacerbating factor of dazzling light and odor, accompanied symptoms of nausea, vomiting, and photophobia, were more apparent in cluster 1 compared to 3. Triptan was more used in cluster 1 compared to cluster 3.

Regarding the burdens for lives, GAD-7 and PHQ-9 were not different among the three groups. However, HIT-6 and MIDAS scores were higher in cluster 1 compared to cluster 3. Regarding baseline MHD, AMD, and treatment outcomes, they were not statistically different among the three clusters (Supplementary Table 1). The initial MHD and AMD were not correlated to HIT-6 nor MIDAS scores by Spearman’s correlation test (Supplementary Table 2).

## 4 Discussion

We conducted a retrospective review of medical records for 821 adult CM patients, and this was the first study to use cluster analysis to categorize CM based solely on their baseline and headache characteristics and to compare the differences in headache burdens and treatment outcomes between clusters.

Cluster analysis revealed three CM types: cluster 1 (female, migraine-like), cluster 2 (older, higher BMI, smoker), and cluster 3 (female, less-migraine-like). Cluster 1 experienced a greater burden of illness than cluster 3. However, initial MHD, AMD, and treatment outcomes showed no statistical differences among the clusters. Despite differences in burden, the years of migraine and treatment sensitivity were not different across clusters.

### 4.1 The possible existence of CM subtypes

There have been a number of papers that have epidemiologically investigated headache characteristics in migraine patients, and several unique characteristics of Japanese migraine have been proven. In a previous Japanese study (1) on CM, the presence of phonophobia (3.8%) and photophobia (0%) were lower than those in the American report (14) (26 and 19%, respectively). Other migraine-like characteristics are also less seen in Japanese CM than in American CM, such as unilateral pain (38% in Japanese CM and 43% of American CM) and pulsating pain (38 and 53%). The migraine-like symptoms are less reported by patients, supposedly due to the Japanese belief that working hard is a virtue (*“gaman”* in Japanese, generally translated as ‘perseverance’, ‘patience’, ‘tolerance’, or ‘self-denial’). The concept of *gaman* may, again, have contributed to these discrepant findings between patients with migraine in Japan and America, with concepts such as ‘tolerance’ and ‘self-denial’ resulting in Japanese patients being less likely to report migraine-related symptoms (1). In addition to such cultural background that Japanese CM patients do not often present migraine-like symptoms, our study showed, using cluster analysis on the questionnaire sheets and medical records, that mathematically, there are two headache-characteristics-based clusters as subtypes of CM; female CM were divided into migraine-like (cluster 1) and less-migraine-like (cluster 3) CMs, suggesting that there are additional subtypes of CM that the ICHD-3 has not still separated.

In addition to clusters 1 and 3, a cluster of patients with higher age, higher BMI, and smoker (cluster 2) was calculated. Cluster 2 was a group not directly related to headache characteristics. Obesity may increase inflammatory mediators, exacerbate migraine attacks, and be a risk for CM development (15). Conversely, long-standing migraine exacerbate obesity (16). Also, the smoking rate is higher in migraine patients, and medication overuse, which causes an MHD increase, is related to smoking (17). Furthermore, migraine and obesity may share genetic predispositions, such as dysfunctions in pathways involving orexins, which modulate both pain and metabolism (15). The older age of the first onset of headache may be related to secondary sexual characteristics and problems specific to adolescence (18). Based on the above, cluster 2, unlike clusters 1 and 3, may be related to a possible mechanism for CM development involving obesity and environmental factors like smoking.

Cluster 2 is primarily defined by higher age, BMI, and smoking status, yet it lacks distinct headache characteristics. This raises a question: Does this cluster represent a true CM subtype, or is it a group influenced by confounding lifestyle factors? Given that Clusters 1 and 3 are defined based on headache characteristics, while Cluster 2 is differentiated primarily by demographic and lifestyle factors, further investigation is needed to determine whether these elements should be considered separately.

### 4.2 Misdiagnosis of CM as CTTH

A large multicenter study revealed that 8% of general practitioners and 35% of headache specialists (of whom 51% were neurologists and/or headache specialists) consulted for migraine and made the correct diagnosis (19). However, as the study itself relies on diagnoses made by one headache specialist, there may still be cases of CM that remain undiagnosed. This also suggests that patients who have fewer migraine-like symptoms, such as those in Cluster 3, may still face a risk of misdiagnosis. In particular, if CM is misdiagnosed as TTH and prophylactic treatments, such as CGRP-related monoclonal antibodies, are not appropriately selected, improvement may be limited. Although educational initiatives on migraine have been introduced for both doctors and patients (20), it is important to recognize that certain patients with CM, particularly those in Cluster 3, may present with fewer migraine-like symptoms, making accurate diagnosis more challenging. Furthermore, considering the potential coexistence of CM and chronic TTH, tailoring treatment choices to the specific characteristics of each patient’s headache—such as combining treatments for both migraine and TTH—may be advisable.

### 4.3 MHD and burden

In general, the more days of headache a migraine patient has, the more difficult it is to live with (1, 14). However, there is still debate over how many days should be the cutoff for the migraine severity. The report indicates no significant difference in disruption to life between migraine with an MHD of 8 or more days and migraine with an MHD of 15 or more days (5). Some papers found no significant difference in the degree of Work Productivity and Activity Impairment (WPAI) when grouped by MHD (1). There were no significant differences in evaluating emotional and functional disability between CM (15 or more MHD) and HFEM (10–14 MHD) (21).

In our CM patients, MHD was not significantly different for each cluster, and HIT-6 and MIDAS showed no correlation trend with MHD nor AMD, suggesting that the MHD and AMD may be inadequate to describe the characteristics and degree of burden. In high-frequency migraine patients with MHD≧8 (5), including CM patients, scales such as HIT-6 and MIDAS may be more important than the number of MHD to determine the degree of disability. It is hoped that scales such as the HIT-6 (22) and MIDAS will become more widely used to evaluate patients’ burdens more accurately, not only using MHD or AMD.

Also, our results indicate that migraine-like CM (cluster 1) has a more severe burden than less-migraine-like CM (cluster 3). Migraine’s bothersome symptom is related to migraine’s burden (23), so migraine-like CM may be more burdensome, compared to TTH-like CM. Apart from the MHD, the intuitive impression of whether a person’s headache is migraine-like or not may be linked to the degree of disability. Further study of the relationship between the number of MHD, MMD, and AMD, the degree of burden, and headache characteristics among CM patients is desirable.

### 4.4 Years of headache disorders

EM sufferers develop CM at the rate of approximately 3% (3) per year. Its risk factors are overuse of acute treatment (3), obesity (24), depression, and stressful life events (4). Also, baseline MHD is related to chronic daily headache development (25). The progression of EM to CM is beginning to be recognized. The cornerstone of this concept is the existence of transformed migraine. Transformed migraine, one of the types consisting of CM in ICHD-3 criteria, is characterized by typical migraine with recurrent attacks in the first 10 to 20 years of age. However, from middle age, the frequency of headaches increases to daily or almost daily. The severity of headaches decreases, and the pain becomes similar to TTH. Photophobia, phonophobia, and nausea become less prominent. However, the migraine component persists, such as exacerbation during menstrual periods and unilateral, pulsating characteristics (26).

Given the progressive nature of migraine, it can be hypothesized that CM with a shorter illness duration may present as more migraine-like, while those with a longer duration may exhibit less migraine-like (more tension-type headache, TTH-like) characteristics. However, in our study, we found no differences between migraine-like and less-migraine-like CMs, both with an average disease duration of around 18 years. While CM is generally viewed as a progressive condition, our results indicate no clear association between headache characteristics and the duration of the disorder. The speed of CM progression may vary among individuals, depending on whether migraine frequency is still increasing or has transitioned to TTH-like symptoms. Further research is needed to determine how controlling risk factors can suppress CM progression and to identify potential biomarkers.

Cluster 2 was older at onset and initial consultation compared to clusters 1 and 3. It remains unclear whether secondary sexual characteristics play a role, or if the interplay of obesity and migraine worsens with age, or whether smoking prevalence increases with age. This cluster is defined by factors unrelated to headache characteristics and may be influenced by external factors that contribute to migraine development.

### 4.5 Treatment for CM

The treatment strategy for CM is early discontinuation of acute medication overuse (or tapering down the overused medication) combined with prophylactic migraine treatment (4, 27). Topiramate, onabotulinumtoxin A, valproate acid, gabapentin, tizanidine, and amitriptyline have been shown as effective prophylactic medications for CM (28). Recently, erenumab, fremanezumab, and galcanezumab are emerging as novel preventative drugs (29). However, there are still few reports on the therapeutic efficacy of CM and its prognostic factors. The favorable factors for reversion to episodic migraine are adherence to migraine prophylactic drugs, lower baseline headache frequency, absence of cutaneous allodynia, physical exercise, and withdrawal of overused migraine abortive drugs (28). There are also reports of psychological (30) and genetic factors (31) related to the prognosis of treatment with erenumab, which cannot be evaluated from the nature of the headache alone.

Our results showed significant variations in burden based on the presence of migraine symptoms, particularly between clusters 1 and 3. However, treatment efficacy did not differ among the clusters. This suggests that CM outcomes may be influenced by factors beyond headache characteristics. While our univariate analysis indicated no differences in treatment response between clusters, this finding may be superficial. A more detailed examination is necessary, considering prophylaxis type and adherence. Additionally, we need to evaluate burden using measures like HIT-6 instead of just tracking MHD, as we lacked this data. In our next study, we plan to record and monitor HIT-6 scores during each patient visit.

### 4.6 Limitations

This study has several limitations. First, it is a retrospective analysis of CM patients diagnosed by a single specialist at one institution, raising questions about generalizability to other settings or non-specialists. Although the initial medical questionnaire was thorough, not all patients were followed up on their treatment, leading to poor adherence, which affects the definitiveness of the treatment outcomes. Adherence was defined in this study as consistent medication continuation throughout the defined follow-up periods. Monitoring of adherence primarily relied on patient-reported data supplemented by clinical notes; however, this method may not capture all nuances of medication compliance accurately. The majority of patients were only followed for 3 months, limiting the ability to assess long-term treatment efficacy. Second, multiple univariate analyses were conducted after the cluster analysis, and while various clustering methods exist, DBSCAN may not always be optimal. Additionally, we intended to explore the prognosis and treatment for each cluster, but found no differences in treatment efficacy.

## 5 Conclusion

Cluster analysis of the 821 CM patients based on headache characteristics identified the potential existence of three distinct types of CM: cluster 1 (predominantly female with CM resembling migraine), cluster 2 (higher age, higher BMI, smoker), and cluster 3 (mostly female with CM that have fewer migraine characteristics). The impact on quality of life was significant in cluster 1 compared to cluster 3. However, there were no statistically significant differences in treatment outcomes, initial MHD, AMD, disease duration, or treatment sensitivity among these clusters.

## Data Availability

The raw data supporting the conclusions of this article will be made available by the authors, without undue reservation.

## Footnotes

^1^ https://www.itoigawa-hp.jp/other/rinri/img/20220926_optout.pdf
