# Supplementary figures and images for: Profiling chronic migraine patients according to clinical characteristics: a cluster analysis approach
Source: Front Neurol. 2025 Mar 10;16:1569333. doi: 10.3389/fneur.2025.1569333 (PMC11932020; doi:10.3389/fneur.2025.1569333)

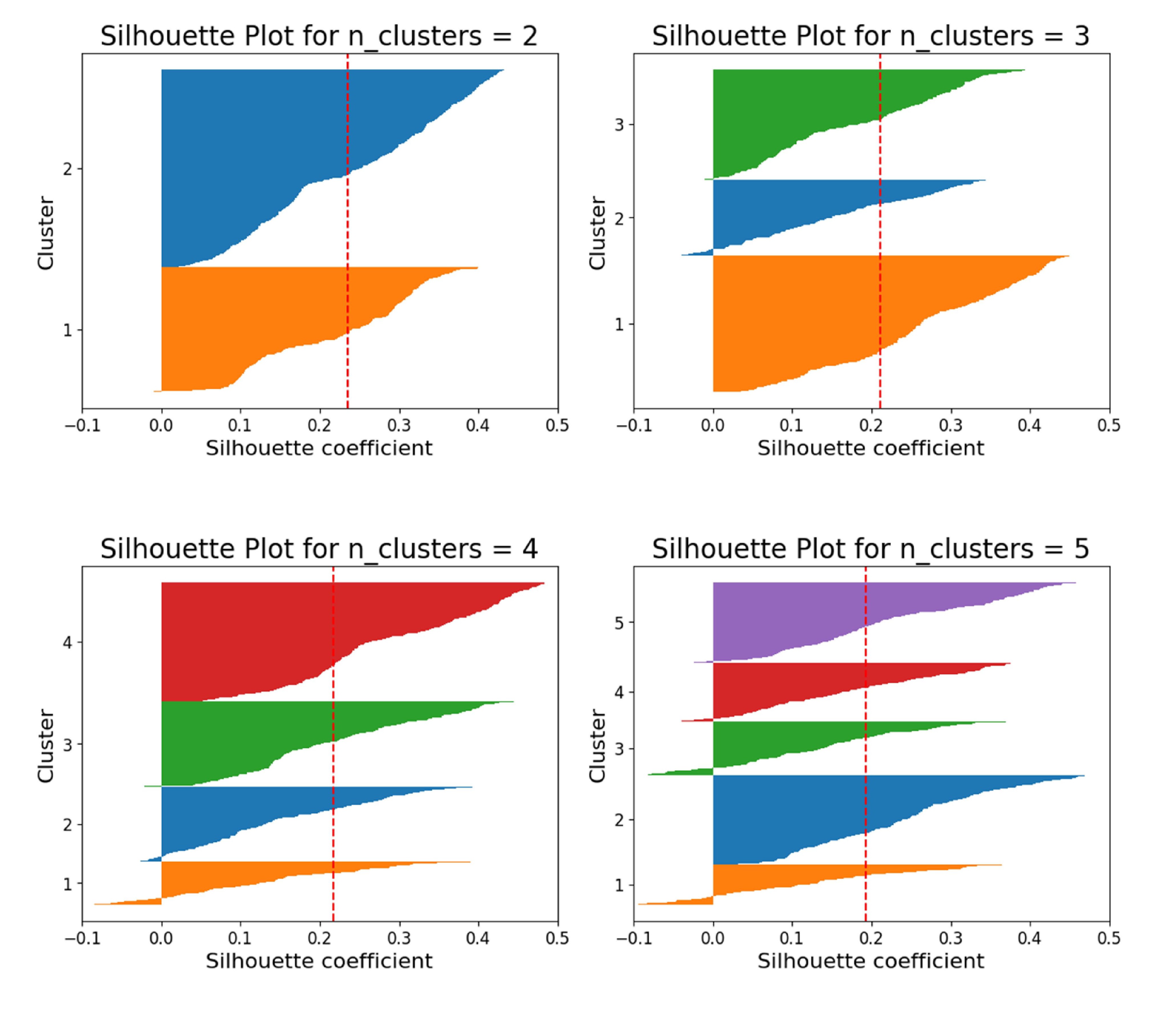

Supplement: Supplementary file 4 [file Image_1.JPEG]
